# The CACNA1C risk allele rs1006737 is associated with age-related prefrontal cortical thinning in bipolar I disorder

**DOI:** 10.1038/tp.2017.57

**Published:** 2017-04-11

**Authors:** M G Soeiro-de-Souza, B Lafer, R A Moreno, F G Nery, T Chile, K Chaim, C da Costa Leite, R Machado-Vieira, M C G Otaduy, H Vallada

**Affiliations:** 1 Mood Disorders Unit (GRUDA), Department of Psychiatry, Institute of Psychiatry, School of Medicine, University of São Paulo (IPq-FMUSP), São Paulo, Brazil; 2 Genetics and Pharmacogenetics Unit (PROGENE), Department of Psychiatry, Institute of Psychiatry, School of Medicine, University of São Paulo (IPq-FMUSP), São Paulo, Brazil; 3 Bipolar Disorder Program (PROMAN), Department of Psychiatry, Institute of Psychiatry, School of Medicine, University of São Paulo (IPq-FMUSP), São Paulo, Brazil; 4 Laboratory of Magnetic Resonance LIM44, Department and Institute of Radiology, University of São Paulo (InRad-FMUSP), São Paulo, Brazil; 5 Experimental Therapeutics and Pathophysiology Branch, Intramural Research Program, National Institute of Mental Health, Bethesda, MD, USA

## Abstract

Calcium channels control the inflow of calcium ions into cells and are involved in diverse cellular functions. The *CACNA1C* gene polymorphism rs1006737 A allele has been strongly associated with increased risk for bipolar disorder (BD) and with modulation of brain morphology. The medial prefrontal cortex (mPFC) has been widely associated with mood regulation in BD, but the role of this CACNA1C polymorphism in mPFC morphology and brain aging has yet to be elucidated. One hundred seventeen euthymic BD type I subjects were genotyped for CACNA1C rs1006737 and underwent 3 T three-dimensional structural magnetic resonance imaging scans to determine cortical thickness of mPFC components (superior frontal cortex (sFC), medial orbitofrontal cortex (mOFC), caudal anterior cingulate cortex (cACC) and rostral anterior cingulate cortex (rACC)). Carriers of the CACNA1C allele A exhibited greater left mOFC thickness compared to non-carriers. Moreover, CACNA1C A carriers showed age-related cortical thinning of the left cACC, whereas among A non-carriers there was not an effect of age on left cACC cortical thinning. In the sFC, mOFC and rACC (left or right), a negative correlation was observed between age and cortical thickness, regardless of CACNA1C rs1006737 A status. Further studies investigating the direct link between cortical thickness, calcium channel function, apoptosis mechanism and their underlying relationship with aging-associated cognitive decline in BD are warranted.

## Introduction

The medial prefrontal cortex (mPFC) is part of a brain circuit involved in emotional expression and mood modulation.^1, 2, 3, 4, 5^ This limbic area displays extensive abnormalities in bipolar disorder (BD).^6, 7, 8, 9, 10^ A single-nucleotide polymorphism on the *CACNA1C* gene, which encodes the calcium v1.2 L-type voltage-gated channel, has been strongly associated with BD.^11, 12, 13^ Moreover, this single-nucleotide polymorphism has been associated with cognitive performance^14, 15^ and regional gray matter (GM) volume changes.^1, 16, 17^ However, there are no studies investigating the influence of the CACNA1C risk allele on mPFC morphology, including cortical thickness or brain aging in BD.

The orbitalfrontal (mOFC) part of the mPFC has widespread connections to the limbic area and adjacent PFC regions.^18^ This same limbic region has also been linked to mood modulation in BD;^19^ for instance, enhanced connectivity between the mPFC and amygdala has been reported in BD patients viewing sad stimuli.^20^ The presence of high connectivity between mPFC and insula, a key region for emotional processing, has also been reported in BD.^21^ Current evidence supports a model of abnormal anatomic connections between PFC and limbic brain structures.^22^

The *CACNA1C* gene encodes to the α1-C subunit of the L-type voltage-gated calcium channel. The influx of Ca^2+^ into neurons activates pathways that rely on Ca^2+^, including neurotransmitter release, calmodulin-dependent protein kinase II and protein kinase C.^23^ The activation of calcineurin inhibits the activity of ionotropic glutamate receptors via receptor desphosphorylation following the Ca^2+^-dependent activation of calmodulin.^24^ Moreover, the activation of calcium v1.2 L-type voltage-gated channel permits cellular influx of calcium following temporary changes in the membrane potential, which activates downstream pathways of genetic transcription, very important to brain plasticity, such as for brain-derived neurotrophic factor.^25, 26^ This way, CACNA1C influx of Ca^2+^ into neurons has neurotrophic properties and works with glutamate ionotropic channels to regulate intracellular Ca^2+^. The CACNA1C risk variant for BD consists of the presence of the A allele of the single-nucleotide polymorphism rs1006737. This single-nucleotide polymorphism is considered a functional polymorphism because its A/A genotype has been associated with greater CACNA1C messenger RNA expression in the PFC compared with G/G or A/G genotypes.^27^ Moreover, it has been reported that BD carriers of the A allele have increased amygdala activity during emotional processing tasks compared to non-carriers. A recent study reported that BD subjects carrying the CACNA1C risk allele A had higher levels of intracellular calcium compared to healthy controls (HCs).^28^

Cortical GM volume is an unspecific measure of brain morphology because it is the product of both cortical thickness and area. Therefore, a decrease in cortical volume may suggest reduced thickness and/or area. The two constituent components of GM result from well-differentiated ontogenic stages during corticogenesis^29^ and appear to have independent genetic etiology.^30^ Cortical neurons are arranged into ontogenetic columns that are located perpendicular to the cortical surface. Studies investigating the impact of CACNA1C on regional brain morphology are less extensive in BD patients than in HCs. In controls, there have been reports of the A allele increasing GM,^16, 31^ brainstem,^17^ right amygdala and right hypothalamus^1^ volumes, whereas reports in BD patients are sparse and have focused on different brain structures.

To the best of our knowledge, the only study investigating the impact of CACNA1C on cortical thickness^32^ in BD type I patients showed negative results for a sample of 71 BD type I patients. Tesli *et al.*^32^ reported no effect of CACNA1C rs1006737 on frontal, parietal, temporal or total cortical thickness.

Thus, the intention of the present study was to investigate whether the presence of CACNA1C risk allele A influences cortical thickness in the main components of the mPFC (mPFC (superior frontal (sFC), mOFC, caudal anterior (cACC) and rostral anterior (rACC) cingulate)).

## Materials and methods

One hundred seventeen (78 females, 18–45 years) BD type I subjects during euthymia were included in this study. Diagnoses were reached by skilled psychiatrists using the Structured Clinical Interview (SCID-I/P)^33^ for DSM-IV TR.^34^ All BD patients had been euthymic for at least 2 months prior to the scanning session. Patients were allowed to be using a combination of antidepressants, lithium, anticonvulsants and antipsychotics according to the clinical criteria without any change on dosage or substance for at least 2 months prior to inclusion in this study. Individuals with neurological disorders or medical disorders, head trauma or current/past (3 months) substance abuse, as well as those who had been treated with electroconvulsive therapy in the last 6 months, were excluded. The Young Mania Rating Scale^35^ and the Hamilton Depression Rating Scale-21 (ref. 36) were used to assess residual subthreshold depressive and manic symptoms. Euthymia was defined as <7 Young Mania Rating Scale and <7 Hamilton Depression Rating Scale-21. The patients also fulfilled the DSM-IV criteria for remission.

The research ethics committee of the Hospital de Clinicas, University of Sao Paulo (CAPPesq), approved the study. Written informed consent was obtained from all study participants.

### Image acquisition

Magnetic resonance imaging was executed using an Intera Achieva 3.0-T system (Philips, Best, The Netherlands) and an eight-channel head coil. Sagittal three-dimensional T1-weighted anatomical images with isotropic 1mm^3^ resolution were obtained with a fast-field echo sequence (TR=7 ms; echo time (TE)=3.2 ms; inversion time (TI)=900 ms; flip angle=8º). Three-dimensional T1-weighted magnetic resonance images were analyzed with the program FreeSurfer v.5.1.0 (The General Hospital, Boston, MA, USA) to obtain volumes for structures, automatically and non-interactively, in right and left hemispheres. Intracranial volume was also measured with the same software for normalization purposes.

Cortical reconstruction and volumetric segmentation were performed with the Freesurfer image analysis suite, which is documented and freely available for download online (http://surfer.nmr.mgh.harvard.edu/). The technical details of these procedures are described elsewhere.^37, 38, 39, 40, 41, 42, 43, 44, 45, 46^ Briefly, this processing includes removal of non-brain tissue using a hybrid watershed/surface deformation technique,^47^ automated Talairach transformation, segmentation of subcortical white matter and deep GM volumetric structures (including hippocampus, amygdala, caudate, putamen and ventricles),^41, 44^ intensity normalization,^48^ tessellation of the GM white matter boundary, automated topology correction,^40, 49^ and surface deformation following intensity gradients to optimally place the gray/white matter and GM/cerebrospinal fluid borders at the location where the greatest shift in intensity defines the transition to the other tissue class.^37, 38, 39^ Once the cortical models are complete, a number of deformable procedures can be performed for further data processing and analysis including surface inflation,^43^ registration to a spherical atlas utilizing individual cortical folding patterns to match cortical geometry across subjects,^50^ parcellation of the cerebral cortex into units based on gyral and sulcal structure,^42, 51^ and creation of a variety of surface-based data including maps of curvature and sulcal depth. This method uses both intensity and continuity information from the entire three-dimensional magnetic resonance volume in segmentation and deformation procedures to produce representations of cortical thickness, calculated as the closest distance from the gray/white boundary to the gray/cerebrospinal fluid boundary at each vertex on the tessellated surface.^39^ The maps are created using spatial intensity gradients across tissue classes and are therefore not simply reliant on absolute signal intensity. The maps produced are not restricted to the voxel resolution of the original data and are thus capable of detecting submillimeter differences between groups. Procedures for the measurement of cortical thickness have been validated against histological analysis^52^ and manual measurements.^53, 54^ Freesurfer morphometric procedures have shown good test–retest reliability across scanner manufacturers and field strengths.^45, 55^

### Genotyping

DNA was obtained from peripheral blood on the day of magnetic resonance imaging exams, according to the salting-out protocol^56^ and then genotyped for CACNA1C rs1006737 using real-time PCR allelic discrimination. PCR amplification for rs1006737 was performed in 5 μl reactions with 5 ng of template DNA, 1 × TaqMan Universal Master Mix (Applied Biosystems, Foster City, CA, USA), 1 × each primer and probe assay, and H_2_O. Thermal cycling consisted of initial denaturation for 10 min at 95 °C, followed by 40 cycles of denaturation at 95 °C for 15 s and annealing at 60 °C for 1 min. Fluorescence detection was performed in the annealing step. Amplification and allelic discrimination were performed on a 7500 Real-Time System (Applied Biosystems). Quality control of Real-time PCR results was done by direct sequencing on an ABI PRISM 3100 Genetic Analyzer (Applied Biosystems). The genotype distribution was in (*χ*^2^=.97) Hardy–Weinberg equilibrium.

### Statistical analysis

Statistical analyses were performed using Stata 13.1 (Statacorp, College Station, TX, USA). Continuous data were described as mean±s.d. if normally distributed. In all analysis, two-tailed values of *P*<0.05 were considered statistically significant. Our first statistical analysis consisted of a linear regression, in which, cortical thickness (left and right sFC, mOFC, cACC and rACC) was the dependent variable, whereas age was the main explanatory variable adjusted for gender. We executed this linear regression separately in two groups: carriers (A/G and A/A) and non-carriers (G/G) of allele A CACNA1C rs1006737. We chose this strategy because our main hypothesis was that the presence of the CACNA1C rs1006737 risk allele A could affect cortical thickness. After that, to investigate the influence of medications in our results, we executed another linear regression in which cortical thickness (left and right sFC, mOFC, cACC and rACC) was the dependent variable, whereas age was the main explanatory variable adjusted for gender and medication status (antidepressants, lithium, anticonvulsants and/or antipsychotics). The regression coefficients (coef) are expressed together with their 95% confidence intervals (CIs).

## Results

Sociodemographic allele distribution information is given in Table 1. Carriers and non-carriers of the A allele did not differ for age, gender, education, dexterity, disease duration or intracranial volume (Table 1), but there was a difference regarding the rate of lithium and antipsychotics use.

CACNA1C A allele carriers (*n*=50) exhibited greater left mOFC thickness (coef=0.08, 95% CI=0.02, 0.14; *P*=.003) compared to non-carriers (*n*=67). No difference in right mOFC thickness was found between carriers and non-carriers of A (coef=0.46, 95% CI=−0.01, 0.11, *P*=0.16). There was no difference between carriers and non-carriers of A in sFC, cACC and rACC cortical thickness (left or right), after controlling for age and gender.

**Figure 1:**
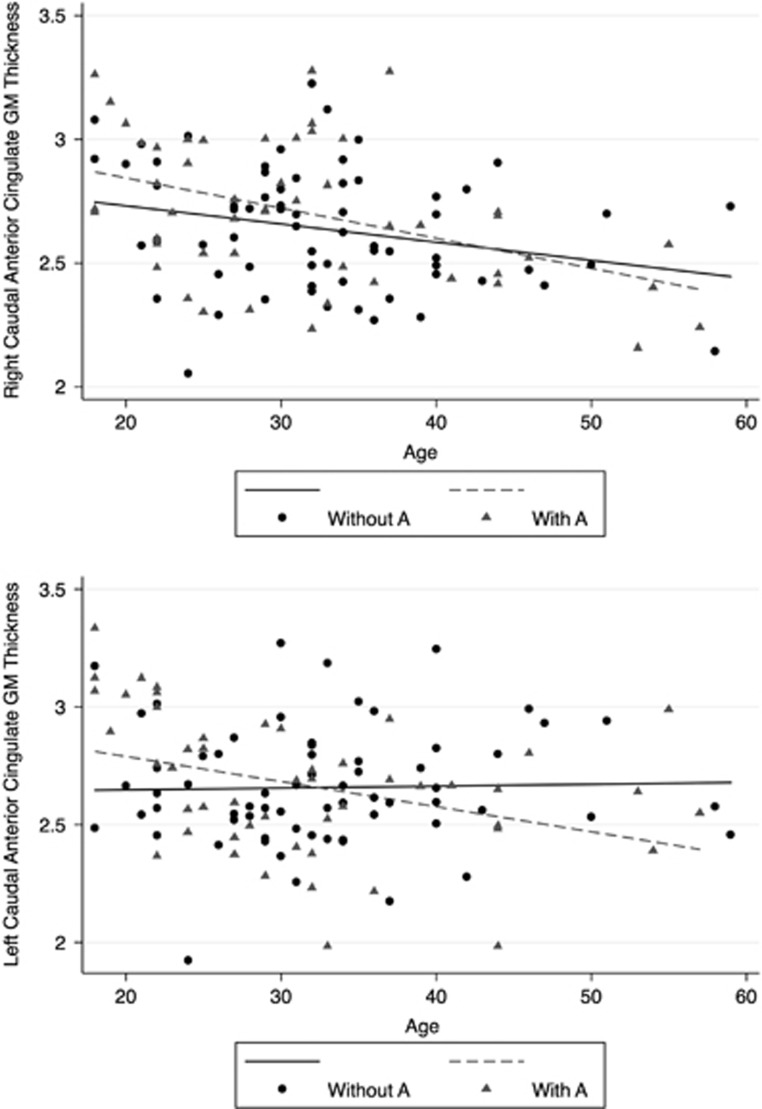
Impact of age on cACC thickness in BD by rs1006737 A allele (corrected by gender and medication status). Right cACC A carriers (*P*=0.003) and A non-carriers (*P*<0.001). Left cACC A carriers (*P*=0.004); A non-carriers (*P*=0.87). BD, bipolar disorder; cACC, caudal anterior cingulate cortex.

Subsequently, we investigated whether the CACNA1C A allele influenced the association between age and cortical thickness. Left cACC thickness negatively correlated with age in A carriers (coef=−0.012, 95% CI= −0.019, −0.004; *P*=.004) but not in non-carriers (coef=0.001, 95% CI=−0.007, 0.008; *P*=0.87; Figure 1). A negative correlation between age and cACC was detected for the right cACC, regardless of CACNA1C A status (A carriers (coef=−0.012, 95% CI=−0.018, −0.006; *P*<.001); non-carriers (coef=−2.2, 95% CI=−0.014, −0.0007; *P*=0.003)).

Finally, we executed a linear regression controlling for medication status (use of antidepressants, anticonvulsants, lithium and/or antipsychotics). Results revealed that among allele A carriers the left cACC cortical thickness remained negatively influenced by age (coef=−0.011, 95% CI=−0.019, −0.004; *P*=0.004) and none of the four classes of medications demonstrated to influence the result. Among allele A non-carriers, there was no impact of age on left cACC cortical thickness (coef=−0.0001, 95% CI=−0.006, 0.006; *P*=0.97), but we observed a positive influence of lithium use (coef=0.14, 95% CI=0.004, 0.28; *P*=0.04) and a negative influence of antipsychotics (coef=−0.15, 95% CI=−0.29, −0.028; *P*=0.0018). The negative association between age and cortical thickness on the right cACC regardless status of A allele CACNA1C rs1006737 remained unchanged after controlling for medication status.

In the sFC, mOFC and rACC (left or right), a negative correlation was observed between age and cortical thickness, regardless of CACNA1C rs1006737 A status or medication use (Supplementary Figure 1).

## Discussion

This study reports two main findings: (1) the influence of the rs1006737 A allele on cortical thickness; and (2) the influence of rs1006737 A allele on the correlation between cortical thickness and age. Euthymic BD I patients who were carriers of the CACNA1C rs1006737 A allele had greater left mOFC thickness compared to non-carriers. Moreover, the risk allele A was selectively associated with left cACC cortical thinning with increasing age, whereas no age-associated cortical thinning was observed in non-carriers.

Our results showing that A allele was associated with increased cortical thickness in left mOFC corroborate previous literature reporting that being a carrier of this allele is associated with greater volume or thickness of several brain structures compared to non-carriers in BD patients and HCs. The impact of the CACNA1C A allele on brain volume in BD type I has previously been evaluated in BD; two studies reported negative results,^32, 57^ whereas two reported increased volume associated with the A allele.^1, 6^ Perrier *et al.*^1^ reported that A carriers had increased GM density in the right amygdala and right hypothalamus in a sample of 41 BD euthymic type I patients compared to 50 HCs. In another study, Frazier *et al.*^6^ (BD type I= 96) reported that A allele carriers had larger bilateral caudate, insula, globus pallidus, frontal pole and nucleus accumbens volumes. The only study investigating the impact of CACNA1C on cortical thickness examined 121 BD patients (71 BD type I, 45 BD type II and 5 NOS) and 219 HC using a 1.5 T scanner and reported no effect of CACNA1C rs1006737 on frontal, parietal, temporal or total cortical thickness.^32^ To our knowledge, our study was the first to exclusively investigate BD type I during euthymia in a group of BD type I patients. We also used a 3 T scanner and focused only on mPFC thickness.

We also report that the CACNA1C A allele influences age-related changes in the mPFC in BD, specifically in the cACC. Allele A carriers demonstrated to have age-related cortical thinning in the left cACC, regardless of medication status. Curiously, the group of subjects not carrying allele A did not have age-related cortical thinning in this region but demonstrated to suffer from a negative impact of antipsychotics and a positive impact of lithium on the left cACC cortical thickness. Cortical thinning is the major manifestation of age-related changes in brain morphology and have been consistently described in both post-mortem and magnetic resonance imaging studies.^58^ These changes include reduction in total brain weight and cortical thickness, as well as gyral atrophy.^58^ Some studies have suggested that changes occur in association areas earlier with lesser changes taking place in primary sensory regions later.^59^ Moreover, these morphological alterations may be occur faster in particular areas of the cortex such as the PFC.^54, 60, 61, 62^ Cortical thickness can be modulated by numerous factors, including the number, size and myelination of neurons in the cortical columns.^63, 64^ For instance, neuronal survival and dendritic volume could be promoted in the adult brain by neurotrophic factors, such as brain-derived neurotrophic factor and glutamatergic signaling via *N*-methyl-d-aspartate receptors,^65, 66^ which have been reported to be altered in BD.^67, 68^ Therefore, the fact that L-type voltage-gated calcium channels control the influx of calcium into neurons and activate calcium-dependent processes, with a key role in neuron plasticity,^23^ might indicate why CACNA1C influences brain morphology. Moreover, it has been described that the use of antipsychotic medication has a negative impact on cortical thickness,^69, 70^ whereas lithium is reported to be associated with increases in cortical and subcortical GM volume,^71, 72^ which is in accordance to our results in non-carriers of CACNA1C allele A rs1006737.

The mechanism by which the A allele influences brain morphology is not fully elucidated, but some data indicate that this may occur through direct regulation of calcium channels activity, neuroplasticity and apoptosis regulation. These molecular changes may result in increased brain volume,^16, 31^ reduced emotional and cognitive processing,^27, 73^ increased brain activation signals during cognitive processing,^27, 73^ and decreased regional connectivity.^16^ The CACNA1C A/A genotype has also been associated with greater CACNA1C messenger RNA expression in the dorsolateral prefrontal cortex than G/G or A/G genotypes.^27^ Another recent study reported that BD patients carrying the CACNA1C allele A had higher levels of intracellular calcium compared to HCs.^28^ Similarly, Yoshimizu *et al.*^74^ reported elevated CACNA1C messenger RNA and greater calcium current density in neurons derived from allele A homozygotes compared to heterozygotes and non-risk (G/G) homozygotes. Higher intracellular calcium causes erratic activation of Ca^2+^-dependent pathways that are normally latent or operate at low levels, causing metabolic imbalances and eventual cell death.^75^ For example, acute raises in intracellular Ca^2+^ may over-activate proteases, lipases, phosphatases and endonucleases that either directly damage cell structure or induce the formation of oxidative-free radicals that mediate cell death.^76^ Taken together, these data support the view that higher activation or larger volumes of brain structures are not synonyms of better functioning. Thus, in a theoretical model, having the CACNA1C A allele would generate neurons with higher intracellular calcium levels, higher excitability and higher activation of calcium-dependent intracellular cascades. This phenomenon in BD patients, that *per se* have intrinsically higher activation of the glutamatergic system,^77, 78^ generates a double risk model for neuron hyperactivation based on glutamate and CACNA1C. Thus, the implications for cognition and age-related cortical thinning fit both models.

In summary, the present findings support a key role for the *CACNA1C* gene polymorphism rs1006737 A allele, previously reported to influence brain morphology and cognition, in the modulation of age-related cortical atrophy in cACC in BD. Our data reinforce the association between CACNA1C and morphological changes probably caused by alterations in neuroplasticity and neuron excitability patterns. Further studies investigating the link between the CACNA1C rs1006737 genotype and the cognitive/brain-morphological phenotype in BD type I are warranted. Moreover, future studies should investigate the rationale for L-type calcium channel antagonists as potential agents for preventing cognitive decline in BD type I patients carrying the CACNA1C A allele.

## Figures and Tables

**Table 1:** Sociodemographic, medication use and prefrontal cortical thickness measures according to the presence of A allele CACNA1C rs1006737

	*BD*		
*rs1006737*	*Without A (*N*=67)*	*With A (*N*=50)*	*Sig. (two-tailed)*
Age	32.7±8.7	31.5±10.2	*P*=0.1
Gender (female/male)	49/18	29/21	*χ*^2^=0.08
Dexterity (*n*; right/left)	58/9	42/8	*χ*^2^=0.69
Education (years)	13.3±3.5	13.1±2.6	*P*=0.3
Illness duration (years)	7.46±7.1	6.18±5.42	*P*=0.35
Lithium use (yes/no)	44/23	42/8	*χ*^2^=0.02
Anticonvulsants use (yes/no)	23/44	14/36	*χ*^2^=0.46
Antipsychotics use (yes/no)	30/37	14/36	*χ*^2^=0.06
Antidepressants use (yes/no)	10/57	4/46	*χ*^2^=0.25
Intracranial volume (mean±s.d.)^a^	1 319 411±279 256	1 317 830±209 098	*P*=0.26
Right mOFC (mean±s.d.)^a^	2.48±0.16	2.54±0.21	*P*=0.01
Right sFC (mean±s.d.)^a^	2.52±0.15	2.56±0.18	*P*<0.001
Right rACC (mean±s.d.)^a^	2.97±0.18	3.01±0.26	*P*=0.08
Right cACC (mean±s.d.)^a^	2.63±0.24	2.70±0.29	*P*=0.86
Left mOFC (mean±s.d.)^a^	2.41±0.16	2.51±0.16	*P*=0.005
Left sFC (mean±s.d.)^a^	2.51±0.15	2.56±0.20	*P*<0.001
Left rACC (mean±s.d.)^a^	2.98±0.19	2.98±0.22	*P*=0.009
Left cACC (mean±s.d.)^a^	2.65±0.25	2.66±0.29	*P*=0.11

Abbreviations: BD, bipolar disorder; cACC, caudal anterior cingulate cortex; mOFC, medial orbitofrontal cortex; rACC, rostral anterior cingulate cortex; sFC, superior frontal cortex.

a Mean and s.d. without adjust for age and gender.
