# Supplementary material for: The CACNA1C risk allele rs1006737 is associated with age-related prefrontal cortical thinning in bipolar I disorder
Source: Transl Psychiatry. 2017 Apr 11;7(4):e1086–. doi: 10.1038/tp.2017.57 (PMC5416698; doi:10.1038/tp.2017.57)
Supplement: Supplementary Figure [file tp201757x1.doc]

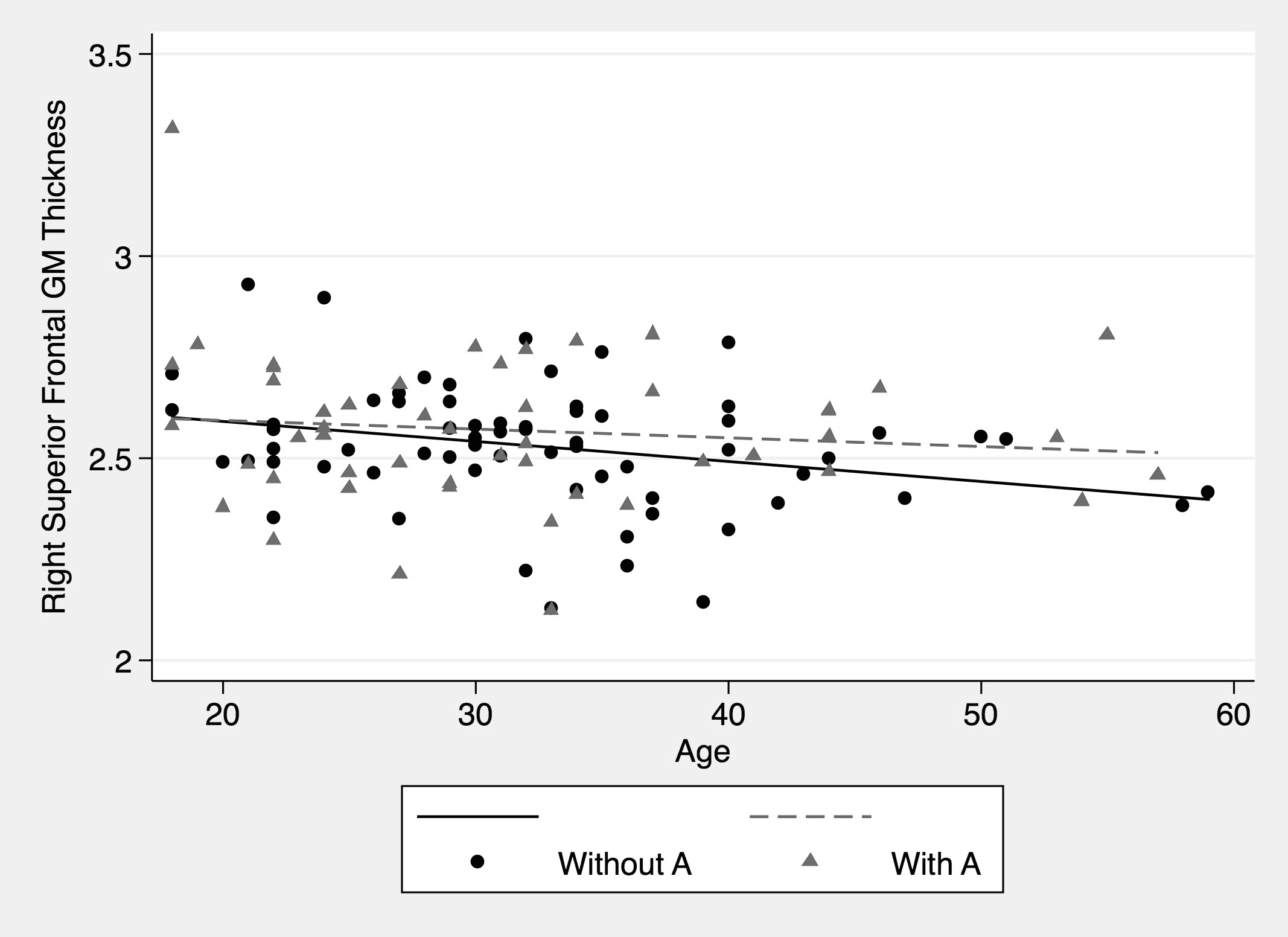

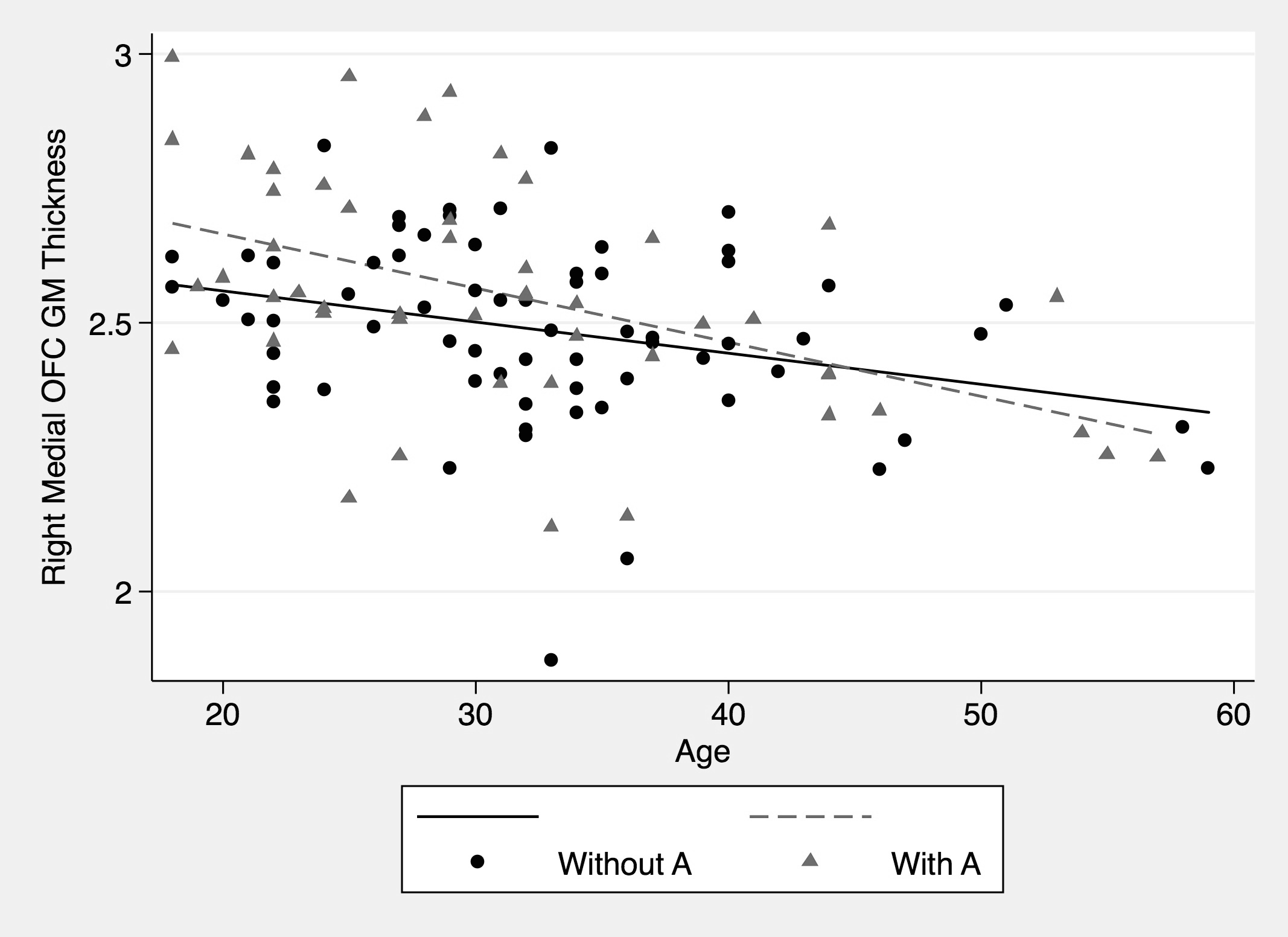

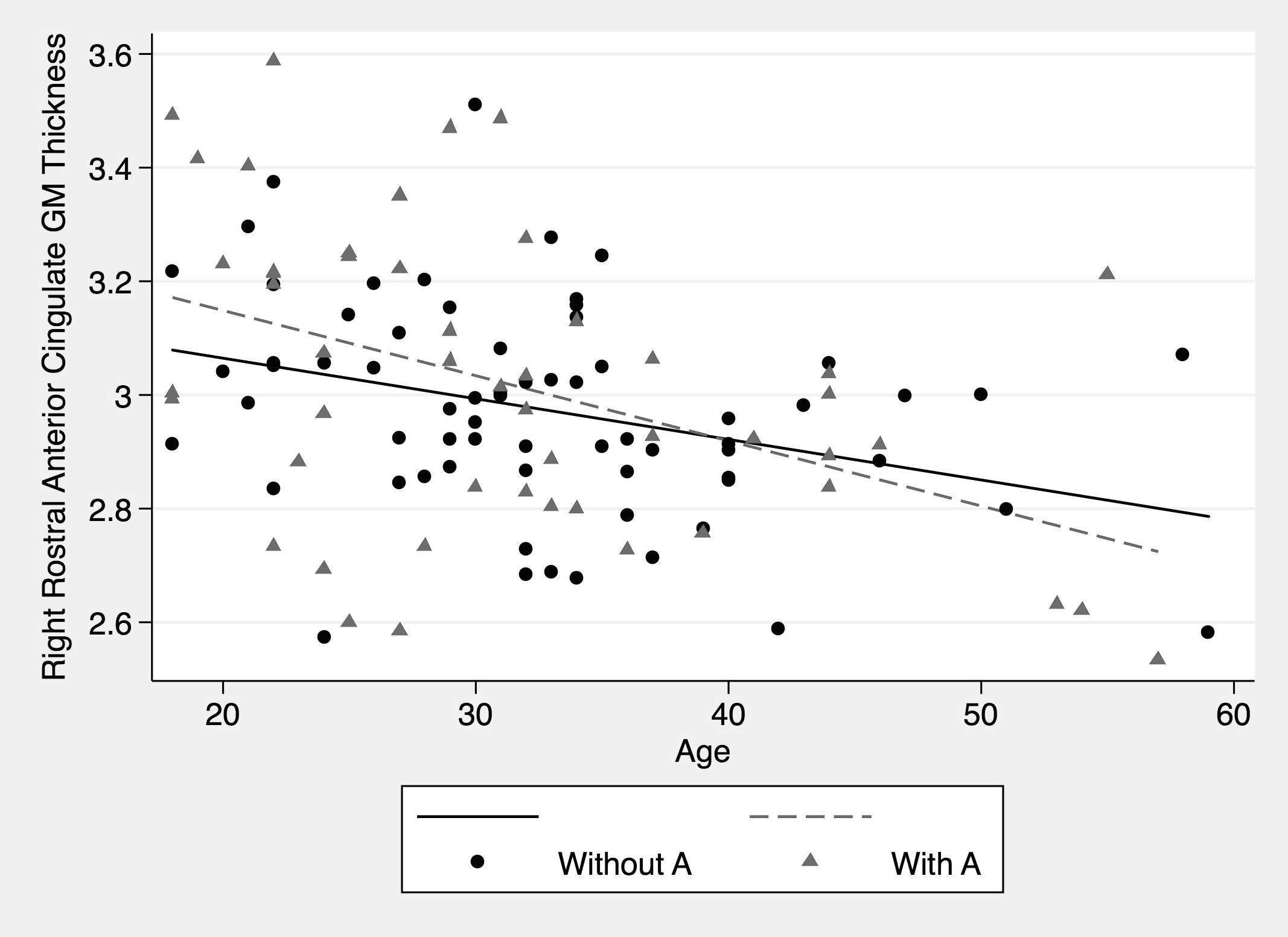

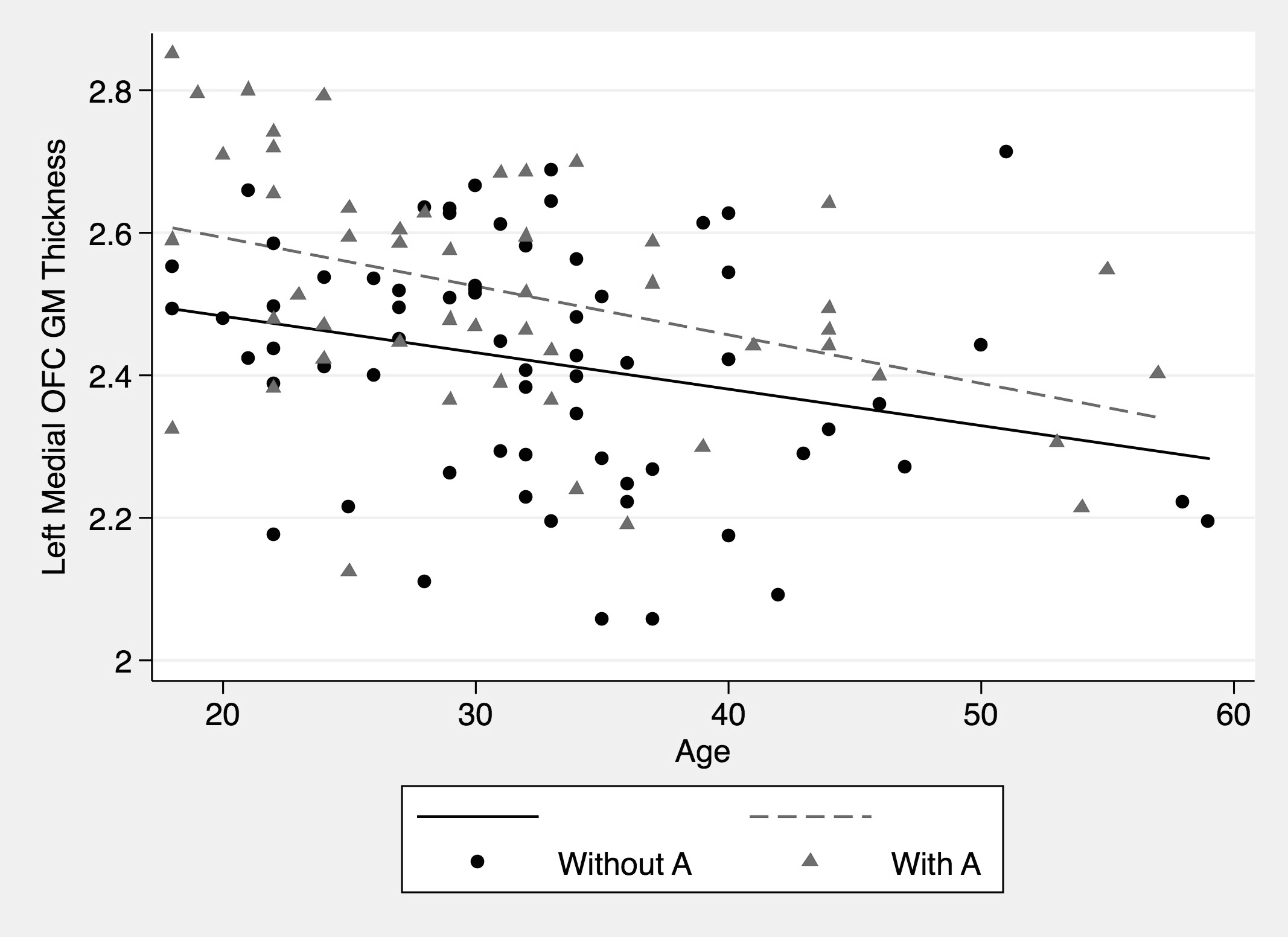

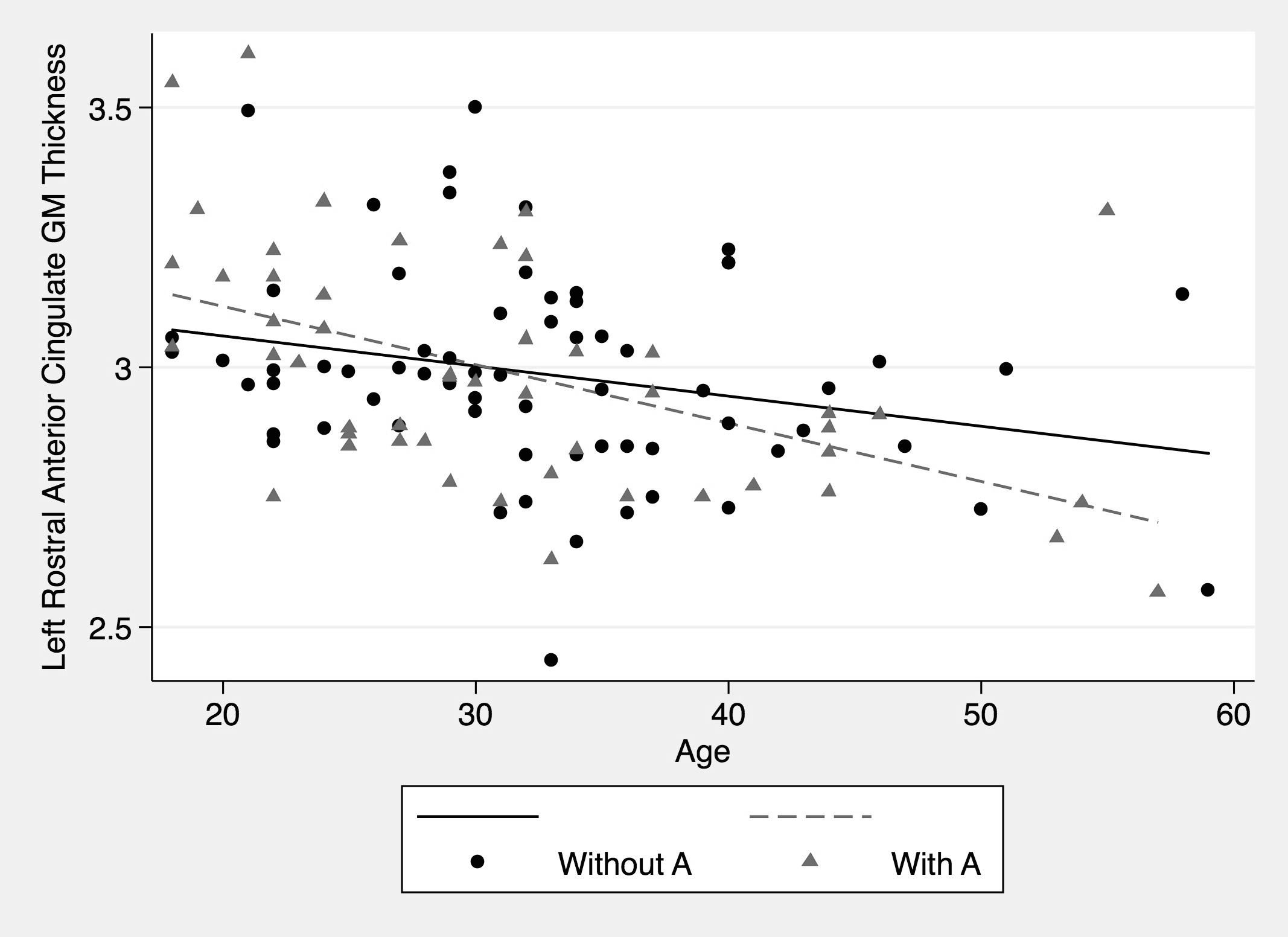

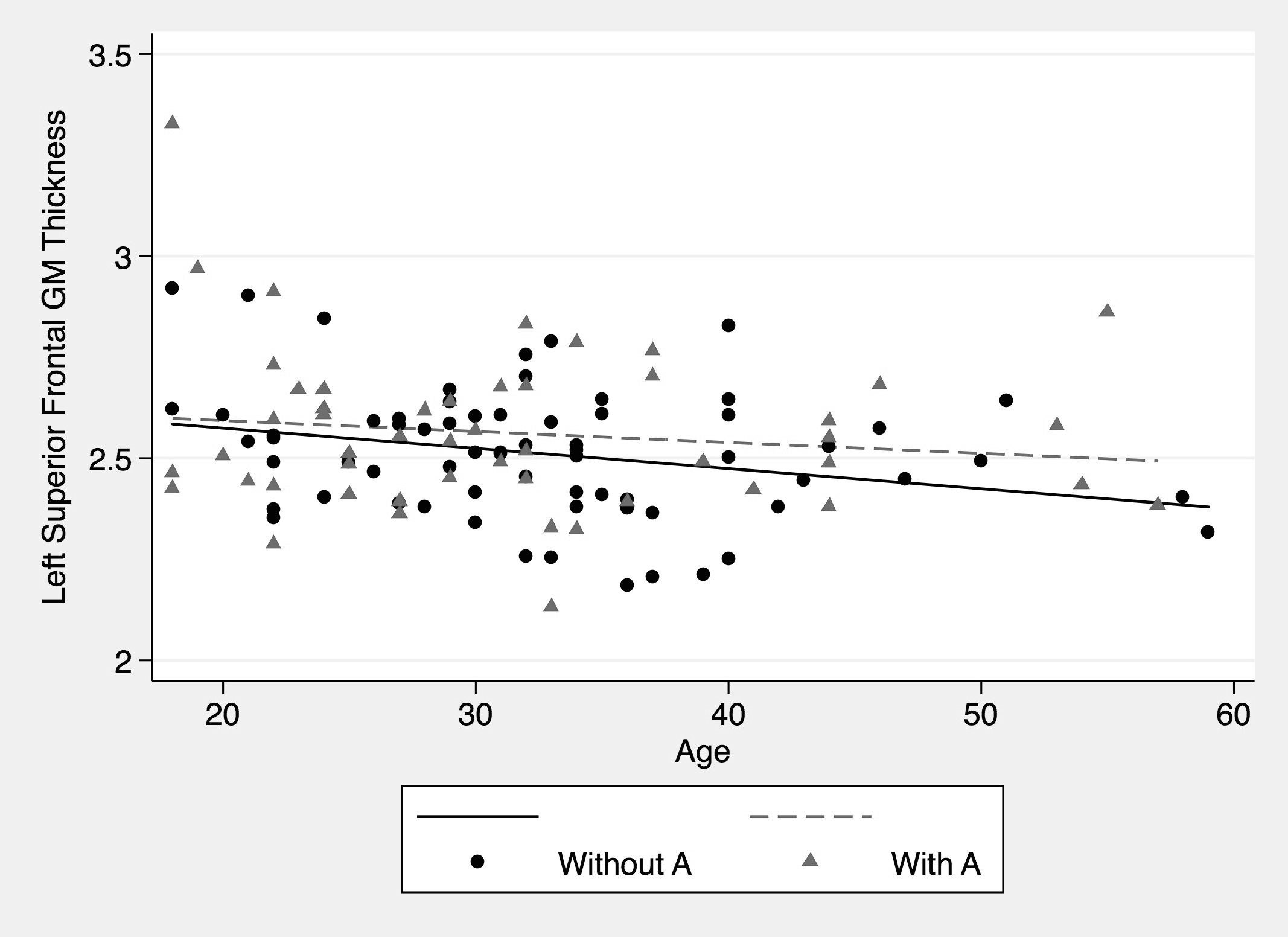
Supplement Figure 1. Age impacts on thickness (corrected by gender) in the left and right rACC, mOFC, sFC regardless of rs1006737 A allele presence.
